# 单克隆B淋巴细胞增多症合并慢性粒-单核细胞白血病继发免疫性血小板减少一例

**DOI:** 10.3760/cma.j.issn.0253-2727.2021.12.019

**Published:** 2021-12

**Authors:** 婧妍 邱, 业钦 沙, 晖 沈, 建勇 李, 华渊 朱

**Affiliations:** 1 南京市浦口区中心医院，江苏省人民医院浦口分院，浦口慢淋中心，南京 211800 Nanjing Pukou Center Hospital, Jiangsu Province Hospital Pukou Branch, Pukou Chronic Lymphocytic Leukemia Center, Nanjing 211800, China; 2 南京医科大学第一附属医院，江苏省人民医院血液科，南京 210029 Department of Hematology, the First Affiliated Hospital of Nanjing Medical University, Jiangsu Province Hospital, Nanjing 210029, China

患者，男，74岁，因“四肢散在出血点进行性加重”于2020年10月就诊于当地医院。血常规：WBC 12.18×10^9^/L，HGB 136 g/L，PLT 1×10^9^/L，淋巴细胞绝对计数（ALC）2.0×10^9^/L，中性粒细胞绝对计数（ANC）7.42×10^9^/L，单核细胞绝对计数（AMC）2.19×10^9^/L，单核细胞百分比（MONO％）18％；全身增强CT提示膈肌上下多发增大淋巴结（最大淋巴结直径11 mm）。外院骨髓免疫表型诊断为B淋巴细胞增殖性疾病伴免疫性血小板减少，予地塞米松及丙种球蛋白治疗，效果不佳。2020年11月患者因黑便5 d转入我院，查体示全身皮肤黏膜散在出血点及紫癜，周身浅表淋巴结及肝脾未及。血常规：WBC 39.12×10^9^/L，PLT 2×10^9^/L，ALC 3.9×10^9^/L，ANC 26.11×10^9^/L，AMC 8.33×10^9^/L，MONO％ 21.3％，HGB 60 g/L；直接抗人球试验阳性，间接抗人球试验阴性；抗核抗体（ANA）阳性（均质型，滴度1∶320）；便常规隐血阳性；凝血功能、抗血小板抗体、LDH、胆红素及网织红细胞未见异常。骨髓示：原始粒细胞6.4％，易见粒系病态造血现象。全片见巨核细胞101个，分类25个，产板巨核细胞1个。外周血涂片：MONO％ 17％，考虑为慢性粒-单核细胞白血病（CMML）-1。骨髓活检提示淋巴细胞小灶增生，CD5（散在+），CD10（−），CD20（++++），PAX5（+++），CD23（−），CyclinD1（−）。骨髓免疫分型提示：淋巴细胞占7.8％，其中异常单克隆B淋巴细胞占49.0％，FS弱表达，表达CD45、CD19、CD20、CD22、CD200、CD305、κ；弱/部分表达：CD5、CD79b、CD38、CD35、CD49d；不表达FMC7、CD81、CD10、CD23、IgM、CD148、CD11c、CD25、CD43、CD103、CD160、Ki-67、CD24、λ；CD34^+^细胞占0.38％，表达髓系标志，其中单核系CD14^+^CD6^−^占94.5％。染色体为正常核型。FISH提示P53缺失、−5/5q−、−7/7q−、20q−、+8均阴性。IGHV突变。淋巴瘤及髓系二代测序均示NRAS突变（p.G13R；50.10％）。患者2020年5月及9月血常规AMC均≥1×10^9^/L、MONO％≥10％，诊断为：单克隆B淋巴细胞增多症（MBL）；CMML；原发免疫性血小板减少症（ITP）。予艾曲波帕口服及输注血小板、止血治疗，2周后患者出血症状好转，但PLT无改善。2020年11月予利妥昔单抗375 mg/m^2^每周×4周方案治疗，首次应用后PLT升至87×10^9^/L，第二次应用后PLT升至正常，而第三次应用后PLT再次下降至2×10^9^/L，复查骨髓仍提示巨核细胞成熟障碍，流式细胞术仍可标记单克隆B淋巴细胞及CD14/CD16表达异常的单核细胞。2020年12月11日起予地西他滨10 mg/d，第1～5天治疗，28 d为一个周期，治疗后AMC逐渐降至正常，并予第4次利妥昔单抗治疗，2021年1月复查PLT为134×10^9^/L，随访2个月PLT均正常，目前仍地西他滨治疗中。

讨论：WHO（2016）MBL的诊断标准为：①B淋巴细胞克隆性异常；②外周血B淋巴细胞<5×10^9^/L；③无肝、脾、淋巴结肿大（所有淋巴结长径均小于1.5 cm）；④无淋巴增殖性疾病的临床特征。本例患者免疫表型为CD19^+^CD5^+^CD23^−^，符合非典型慢性淋巴细胞白血病（CLL）样MBL。患者外周血AMC≥1×10^9^/L、MONO％≥10％、骨髓涂片可见6.4％原始细胞，符合CMML诊断。患者PLT减低，骨髓细胞形态学可见巨核细胞增加伴成熟障碍，符合ITP诊断。初期针对ITP治疗效果不佳，予利妥昔单抗兼顾ITP及MBL治疗，PLT先升后降，去甲基化药物地西他滨对CMML有一定疗效，且在ITP患者中总体有效率可达50％，予该患者小剂量地西他滨治疗后AMC下降、PLT恢复至正常，考虑患者ITP的发生可能与CMML相关。此类病例较为罕见，如临床忽略AMC异常可导致漏诊，我们采用利妥昔单抗联合地西他滨方案取得较好疗效。

